# Role of Poly(A)-Binding Protein Cytoplasmic 1, a tRNA-Derived RNA Fragment-Bound Protein, in Respiratory Syncytial Virus Infection

**DOI:** 10.3390/pathogens13090791

**Published:** 2024-09-12

**Authors:** Devin V. Davis, Eun-Jin Choi, Deena Ismail, Miranda L. Hernandez, Jong Min Choi, Ke Zhang, Kashish Khatkar, Sung Yun Jung, Wenzhe Wu, Xiaoyong Bao

**Affiliations:** 1 Department of Pediatrics, University of Texas Medical Branch, Galveston, TX 77555, USA; dvdavis@utmb.edu (D.V.D.); jinyjiny014@gmail.com (E.-J.C.); deismail@utmb.edu (D.I.); milherna@utmb.edu (M.L.H.); kezhang@utmb.edu (K.Z.); kakhatka@utmb.edu (K.K.); 2 Lester and Sue Smith Breast Center, Baylor College of Medicine, Houston, TX 77030, USA; jongmin.choi@bcm.edu; 3 Verna and Marrs McLean Department of Biochemistry and Molecular Pharmacology, Baylor College of Medicine, Houston, TX 77030, USA; syjung@bcm.edu; 4 Institute of Translational Science, University of Texas Medical Branch, Galveston, TX 77555, USA; 5 Institute for Human Infections & Immunity, University of Texas Medical Branch, Galveston, TX 77555, USA

**Keywords:** tRF, PABPC1, RSV, viral gene transcription and viral genome replication

## Abstract

Respiratory Syncytial Virus (RSV) is a significant cause of lower respiratory tract infections (LRTI) across all demographics, with increasing mortality and morbidity among high-risk groups such as infants under two years old, the elderly, and immunocompromised individuals. Although newly approved vaccines and treatments have substantially reduced RSV hospitalizations, accessibility remains limited, and response to treatment varies. This underscores the importance of comprehensive studies on host–RSV interactions. tRNA-derived RNA fragments (tRFs) are recently discovered non-coding RNAs, notable for their regulatory roles in diseases, including viral infections. Our prior work demonstrated that RSV infection induces tRFs, primarily derived from the 5′-end of a limited subset of tRNAs (tRF5), to promote RSV replication by partially targeting the mRNA of antiviral genes. This study found that tRFs could also use their bound proteins to regulate replication. Our proteomics data identified that PABPC1 (poly(A)-binding protein cytoplasmic 1) is associated with tRF5-GluCTC, an RSV-induced tRF. Western blot experimentally confirmed the presence of PABPC1 in the tRF5-GluCTC complex. In addition, tRF5-GluCTC is in the anti-PABPC1-precipitated immune complex. This study also discovered that suppressing PABPC1 with its specific siRNA increased RSV (-) genome copies without impacting viral gene transcription, but led to less infectious progeny viruses, suggesting the importance of PABPC1 in virus assembly, which was supported by its interaction with the RSV matrix protein. Additionally, PABPC1 knockdown decreased the production of the cytokines MIP-1α, MIP-1β, MCP-1, and TNF-α. This is the first observation suggesting that tRFs may regulate viral infection via their bound proteins.

## 1. Introduction

Respiratory Syncytial Virus (RSV) is a negative-sense, single-stranded, enveloped RNA virus belonging to the *Pneumovirus* genus. RSV is a significant cause of lower respiratory tract infections (LRTI) across nearly all demographics, with heightened mortality and morbidity in high-risk populations such as infants under two years old, the elderly, and immunocompromised individuals. From 2009 to 2019, RSV was the leading cause of hospitalizations for children under the age of one in the United States [1,2,3]. Moreover, RSV significantly contributes to hospitalizations among older adults [4,5]. Increased hospitalizations, intensive care unit admissions, and higher mortality rates due to RSV infection are common among immunocompromised patients [6]. Additionally, outpatient visits due to RSV infection are considerable, demonstrating the substantial medical burden associated with RSV infection.

A significant milestone in RSV disease control was the launch of FDA-approved RSV vaccines in late 2023. While these vaccines have successfully prevented and reduced the symptoms of RSV, there is still room for improvement. A substantial portion of the population still experiences hospitalization and does not respond well to vaccination [7,8]. Vaccines are only available to those aged 60 and older or to newborns via maternal vaccination at 32–36 weeks of pregnancy, with the protection efficacy waning over time [9,10]. These issues highlight the need for effective treatments. Regarding treatments, the FDA-approved monoclonal antibody nirsevimab is designed to protect infants and some young children at an increased risk of severe RSV disease [11]. Despite these significant milestones in RSV prophylactic and therapeutic strategies, there is an urgent need to develop cost-effective treatments with long-lasting efficacy. A comprehensive understanding of the molecular mechanisms underlying RSV replication and the associated host responses will greatly facilitate meeting this need.

Advancements in high-throughput RNA sequencing technology have greatly facilitated the discovery of non-coding RNA (ncRNAs). The identification of tRNA-derived RNA fragments (tRFs) initially faced delays due to enriched tRF modifications disrupting barcoding and sequencing read-through [12], but they were soon recognized as functional molecules after their discovery. For instance, tRFs have demonstrated significant roles in regulating cell proliferation, migration, and invasion in various cancers [13]. Additionally, they are implicated in diverse mechanisms underlying neurodegenerative diseases and aging [14,15,16]. Our research and that of our colleagues have further highlighted their functional importance in viral infections [17,18,19,20]. Following the discovery of RSV-induced tRFs, our focus has been on elucidating their specific functions and associated mechanisms. Previously, we demonstrated that a tRF derived from the 5′ end of mature glutamic acid (Glu) tRNA, with a CTC codon (tRF5-GluCTC), promotes RSV replication by targeting the mRNA encoding APOER2 to release the RSV P protein from the APOER2-P complex [21]. However, whether tRF5-GluCTC regulates replication through targeting protein remains to be determined.

To address this question, we carried out a proteomics study using synthesized biotinylated tRF5-GluCTC as bait, followed by using streptavidin beads to pull down the tRF5-GluCTC complex to identify bound proteins, as described previously [22]. Among the proteins that showed a greater enrichment in the tRF5-GluCTC complex compared to scrambled oligos (fold enrichment ≥10), we selected poly(A)-binding protein cytoplasmic 1 (PABPC1) as the primary target of our studies due to its known involvement in other viral infections [23,24,25,26]. We then conducted extensive experiments to validate the interaction between PABPC1 and tRF5-GluCTC and to investigate its impact on RSV infection.

## 2. Materials and Methods

### 2.1. Cell Cultures and Virus Preparation

A549, human alveolar type II-like epithelial cells, and HEp-2, human epithelial type 2 cells, were obtained from ATCC, Manassas, VA, USA. A549 cells were maintained in the F12K medium, and HEp-2 cells were maintained in the minimal essential medium. Both media were supplemented with 10% (vol/vol) FBS, 10 mmol/L glutamine, 100 IU/mL penicillin, and 100 µg/mL streptomycin, as previously described [21,27]. The RSV long strain was grown in HEp-2 cells and purified by sucrose gradient, as described [28,29]. The viral titer was determined by immunostaining in HEp-2 cells using polyclonal biotin-conjugated goat anti-RSV antibody (7950-0104; Bio-Rad, Hercules, CA, USA) and streptavidin peroxidase polymer (Sigma, St Louis, MO, USA) sequentially, as described [28,29].

### 2.2. Preparation of Cytosolic Fraction

The cytosolic fraction was prepared as described by Kwanbok Lee et al. [30]. In brief, A549 cells were seeded on 15 cm dishes. Once the cells reached approximately 90% confluence, they were harvested and resuspended in 1 mL of lysis buffer (50 mM of Tris-Cl [pH 7.4], 1.5 mM of MgCl_2_, 75 mM of NaCl, 0.5% Nonidet P-40) containing 10 mM of ribonucleoside-vanadyl complex (New England Biolabs, Ipswich, MA, USA) and protease inhibitor cocktail (Calbiochem, San Diego, CA, USA). The cell lysates were centrifuged at 6000× *g* for 10 min at 4 °C. The supernatant was then further centrifuged at 100,000× *g* for 1 h at 4 °C to remove nuclear fractions.

### 2.3. tRF-GluCTC-Bound Proteins Identification by Proteomics

Then, 1 mg of streptavidin magnetic beads (New England Biolabs, Catalog #S1420S) was washed twice with 0.5 mL of Buffer A150 (10 mM of HEPES [pH 7.9], 1.5 mM of MgCl_2_, 150 mM of KCl, 0.5 mM of dithiothreitol) containing RNase inhibitor (New England Biolabs) and protease inhibitor cocktail (Calbiochem). Next, 1 nmol of biotinylated synthetic control (Bio-tRF5-CN) or tRF5-GluCTC mimic RNA oligos (Bio-tRF5-GluCTC) (Sigma, Woodland, TX, USA) was incubated with pre-washed streptavidin magnetic beads in 0.25 mL of Buffer A150 for 30 min at 4 °C, followed by washing twice with 0.5 mL of Buffer A150. The immobilized biotinylated RNA on streptavidin beads was incubated with uninfected A549 cell lysate for 30 min at 4 °C and followed by washing with 1 mL of Buffer A150 once and 0.5 mL twice. After washing with 1 mL of 1X PBS once, the bead–protein complex was frozen and stored at −80 °C in a freezer until being subjected to mass spectrometry analyses using a nanoLC-1000 (Thermo Scientific, Waltham MA, USA) coupled to an Orbitrap Fusion mass spectrometer (Thermo Scientific) with ESI source and the subsequent identification of proteins using the Proteome Discoverer 2.1 interface (PD 2.1, Thermo Scientific) with the Mascot algorithm (Mascot 2.4, Matrix Science). The intensity-based absolute quantification (iBAQ) value was computed via dividing the sum of peptide intensities by the number of theoretically observable peptides in the protein.

### 2.4. Validation of PABPC1 in tRF5-GluCTC Complex

The pulldown complex using a biotinylated synthesized tRF5-GlyCTC as a bait, followed by streptavidin beads, was harvested similarly to as described above. Scrambled oligos were used as controls. The complex was then suspended in SDS sample buffers, and a western blot assay was performed using anti-PABPC1 antibody (sc-32318, Santa Cruz Biotechnology, Dallas, TX, USA). A small aliquot was saved for input assessment.

### 2.5. Immunoprecipitation

A549 cells were infected with RSV at MOI = 1; uninfected cells were used as mock. At 30 h post-infection, the cells were subjected to UV cross-link. Cells were washed with PBS. Then, the cell lysate was harvested using 1.5 mL of lysis buffer 1 from immunoprecipitation kit (protein G) (Cat #11719386001, Roche, IN, USA) with RNase inhibitor. The cell lysates were then incubated with the anti-PABPC1 antibody for 4 h at 4 °C overnight, followed by incubation with IgG beads, which were extensively washed with lysis buffer 1 three times at 4 °C. The immunoprecipitants were prepared according to the manufacturer’s instructions. The RNAs in the PABPC1 immune complex were extracted using Trizol LS for qRT-PCR to quantify tRF5-GluCTC. In this experiment, cell lysates without anti-PABPC1 antibody treatments were used as negative controls, and an aliquot was saved for input assessment.

### 2.6. siRNA Transfection and Viral Infection

A549 cells, grown in a six-well plate with 80–90% confluence, were changed with 1.5 mL/well fresh growth media right before the transfection. For each well, 8 µL of transfection reagent lipofectamine 2000 was mixed with siRNAs in 500 µL of OptiMEM. The mixtures containing siRNAs with a final concentration of 100 nM were then added to cells. At 24 h post-transfection, RSV at an MOI of 1 was inoculated into cells in FK-12 medium with 2% FBS and 1% P/S. After 2 h of incubation, the medium was removed and replaced with fresh FK-12 medium with 2% FBS and 1% P/S after being washed two times with PBS. At 15 h post-infection, the samples were harvested for viral titration and qRT-PCR.

### 2.7. RNA Extraction and qRT-PCR

After RSV or mock infection, cells were washed with PBS. 1 mL/well of Trizol (Thermo Scientific) was then added to a 6-well plate to harvest RNAs according to the manufacturer’s instructions. To confirm the PABPC1 knockdown, RNAs were subjected to the reverse transcription into cDNA using a iScript™ cDNA Synthesis Kit (Bio-Rad, Hercules, CA, USA) according to the manufacturer’s instructions. qRT-PCR was performed using iTaq™ Universal SYBR Green Supermix (Bio-Rad) with primers specific to PABPC1 in the CFX Connect Real-Time PCR System (Bio-Rad). To quantify the viral genome or genes of RSV, total cellular RNA was extracted, and cDNA was synthesized using RSV-specific reverse transcription primers with TaqMan™ Reverse Transcription Reagents from Thermo Fisher Scientific, followed by qRT-PCR, as described in [31,32]. The expression of RSV nucleoprotein N or RNA-dependent RNA polymerase L was calculated by the 2^−ΔΔct^ method with normalization by β-actin expression. The amount of RSV genome was calculated using the absolute quantitation method. Information about the primers used for reverse transcription and the qRT-PCR is shown in Table 1.

### 2.8. Virus Titration Assay

RSV harvested from A549 cell lysates was diluted in a 3-fold serial dilution, followed by seeding 50 µL of them to Hep-2 cells, grown confluent in 96-well plates. The completed plate was then incubated at 37 °C for 1 h. Then, 100 µL of MEM with 2% serum and 0.75% methylcellulose was placed in each well and incubated for two days. Immunostaining was then performed to quantify the infectious particles as described [28,29]. In brief, cells on day two post-infection (p.i.) were fixed and washed three times with PBS. The fixing buffer was removed, and the plate was washed three times with PBS containing 1% BSA. Subsequently, the viral titer was determined by immunostaining in Hep-2 cells using polyclonal biotin-conjugated goat anti-RSV antibody (Cat#: 7950–0104, Bio-Rad) and streptavidin peroxidase polymer (Cat# S2438, Sigma-Aldrich, St. Louis, MO, USA) sequentially.

### 2.9. Statistical Analysis

The experimental results were analyzed using GraphPad Prism 5 software. An unpaired two-tailed *t*-test was employed to compare the difference. A *p* value < 0.05 was considered to indicate a statistically significant difference. Single and two asterisks represent a *p*-value of <0.05 and <0.01, respectively. Means ± standard errors (SE) are shown.

## 3. Results

### 3.1. PABPC1 Interacts with tRF5-GluCTC

**Figure 1:**
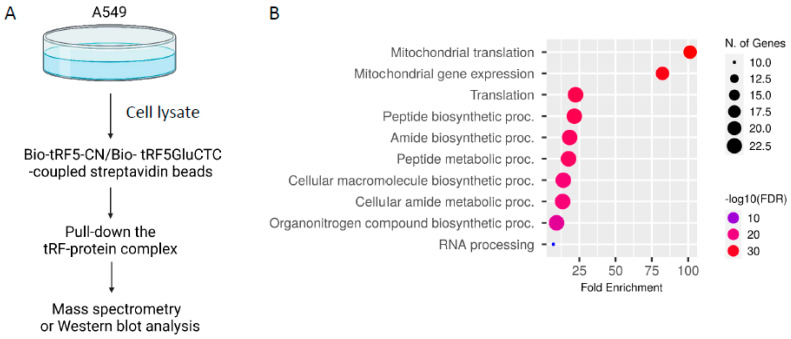
(**A**) The schematic workflow used to identify tRF5-GluCTC-associated proteins in the cell lysates from uninfected A549 cells. (**B**) Go enrichment analysis of the tRF5-GluCTC-bound proteins.

To determine whether tRF5-GluCTC regulates host responses to RSV infection through bound proteins, we mixed biotinylated tRF5-GluCTC mimic (Bio-tRF5-GluCTC)/streptavidin beads with a cytosolic fraction of uninfected A549 cells, followed by a proteomics study (Figure 1A). Biotinylated scrambled RNA oligos (Bio-tRF5-CN) were used as controls. The sequences of synthesized oligos are listed in Table 1. In brief, the bead–protein complex was washed with the same buffer containing 300 mM of KCl, and bound proteins were eluted by increasing the salt concentration (0.6 M and 1.2 M KCl in the same buffer). The eluted proteins were subjected to mass spectrometry analyses for identification. Compared to the control mimic dataset, we identified 31 proteins bound explicitly to tRF5-GluCTC (Table 2). The GO enrichment analysis indicated that these 31 genes were enriched in protein translation, the peptide metabolic process, and RNA processing terms (Figure 1B).

**Figure 2:**
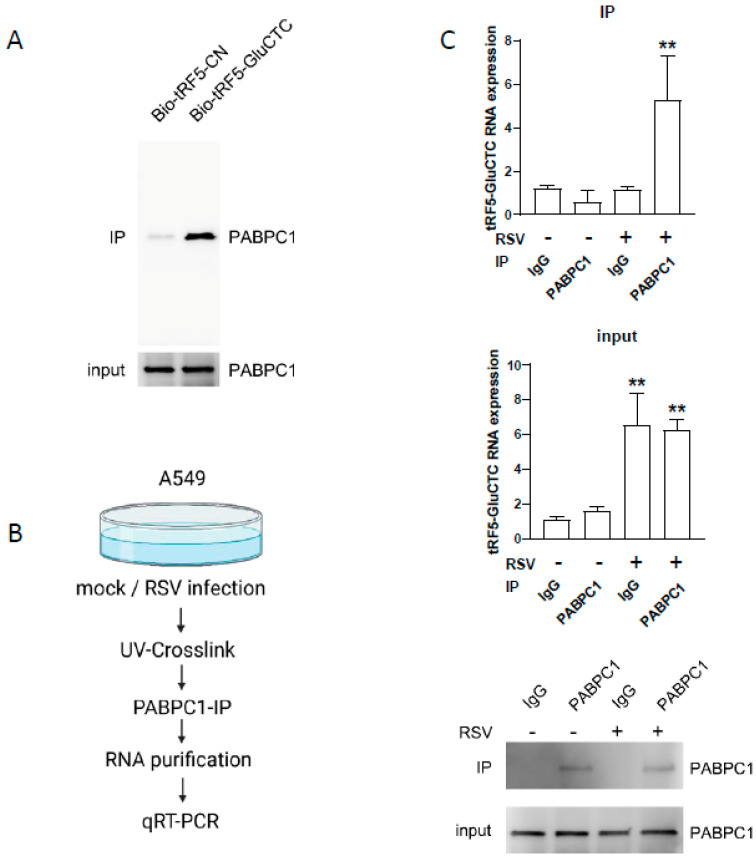
Experimental Validation of tRF5-GluCTC-PABPC1 interaction. (**A**) The presence of PABPC1 in the pulldown complex of Bio-tRF5-GluCTC was revealed by western blot using an anti-PABPC1 antibody. (**B**) The schematic workflow used to detect RSV-induced tRF5-GluCTC in the PABPC1 pulldown complex by qRT-PCR. (**C**) tRF5-GluCTC in the PABPC1 pulldown complex was quantified by qRT-PCR, and western blots were performed to validate proper PABPC1 immunoprecipitation and PABPC1 input. ** *p* ≤ 0.01, compared to the igG RSV group (upper panel of (**C**)), or compared between the first and third columns or the second and fourth columns (lower panel of (**C**)).

PABPC1 was selected for experimental confirmation due to its reported role in viral infections [23,24,25,26]. A Bio-tRF5-GluCTC-protein complex was prepared as described above, and the proteins were suspended in SDS sample buffer. PABPC1 was then detected by western blot using a specific antibody, similar to the method described in reference [22]. The input was also checked. As shown in Figure 2A, PABPC1 was present in the tRF5-GluCTC complex.

We also investigated the PABPC1-tRF5-GluCTC interaction in mock- or RSV-infected cells. In brief, mock- or RSV-infected cells were harvested at 30 h p.i. Before adding the lysis buffer, the cells were UV crosslinked. PABPC1 antibody was then mixed with cell lysis, followed by the IgG bead pull-down and tRF5-GluCTC detection by qRT-PCR (Figure 2B), similar to what we previously described [28]. As shown in Figure 2C, tRF5-GluCTC was induced by RSV infection in A549 cells, and induced tRF5-GluCTC was detected only in RSV-infected and PABPC1-antibody-treated cells, supporting the PABPC1-tRF5-GluCTC interaction. Western blot analysis confirmed the expected enrichment of PABPC1 by IP from mock and infected cell lysis, while the PABPC1 input was comparable among all samples (Figure 2C).

### 3.2. The Impact of PABPC1 on RSV Infection

**Figure 3:**
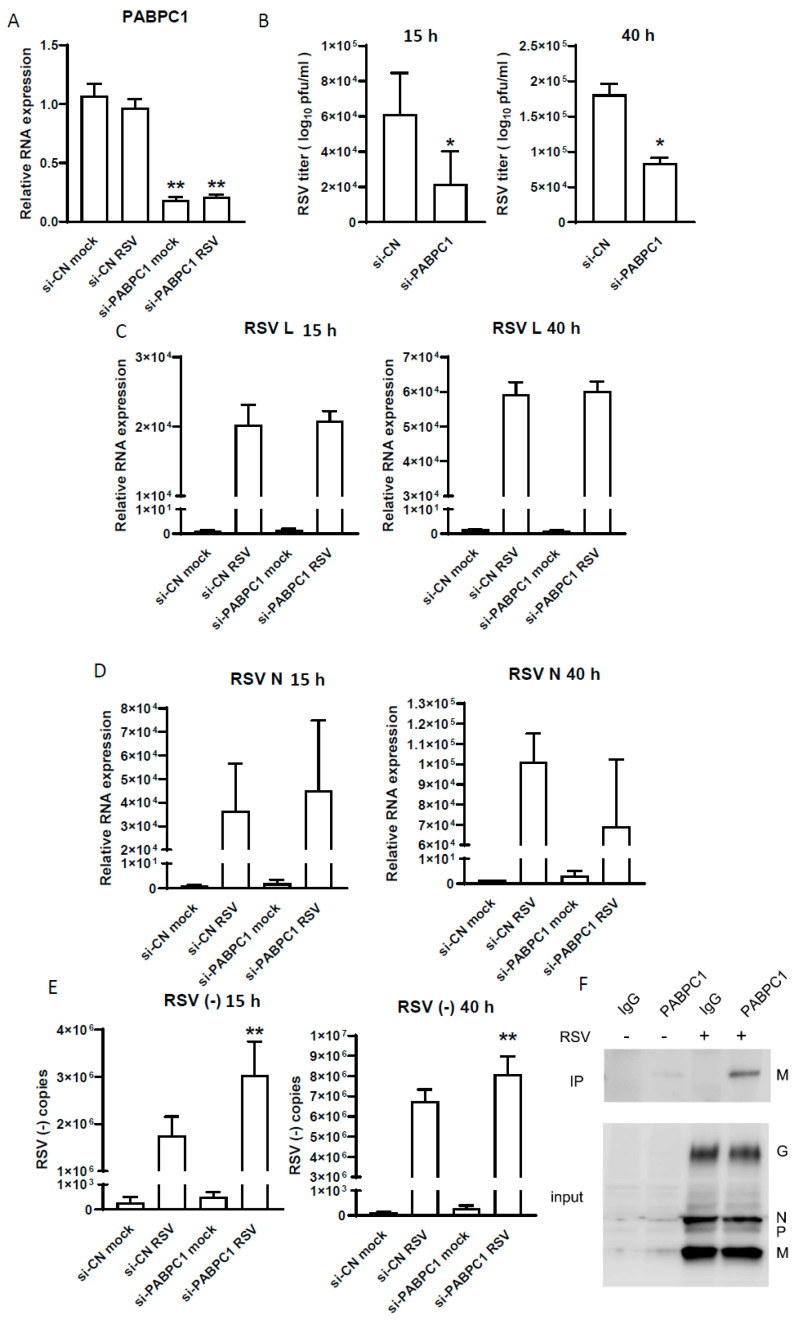
The impact of PABPC1 on RSV infection. A549 cells were treated with 100 nM of si-PABPC1 or si-cn. At 24 h post-transfection, cells were mock infected or infected with RSV at an MOI of 1. After 2 h of absorption, the inoculation was removed and fresh medium was supplied. Cells were harvested at 15 h or 40 h p.i. for RNA preparation or infectious particle quantification. (**A**). The knockdown of PABPC1 by its specific siRNAs was confirmed by qRT-PCR. (**B**) The impact of PABPC1 knockdown on the production of progeny virus. (**C**,**D**) The effect of PABPC1 on the expression of RSV long protein L (**C**) and nucleoprotein N (**D**). The relative viral gene expression was normalized with β-actin. (**E**) The impact of PABPC1 on viral genome synthesis. (**F**) The RSV matrix protein M is present in the PABPC1 pulldown complex. * *p* ≤ 0.05, ** *p* ≤ 0.01, compared to the si-CN RSV group.

To test whether PABPC1 is functional in controlling RSV infection, siRNA specific to PABPC1 was employed to silence its expression in A549 cells, followed by RSV infection at MOI = 1 for 15 h; the production of progeny virus was determined by titration. The qRT-PCR results, shown in Figure 3A, demonstrated successful PABPC1 downregulation by its specific siRNAs. As shown in Figure 3B, significantly fewer infectious particles were observed in PABPC1-siRNA-treated (si-PABPC1) cells compared with the cells treated with scrambled control siRNAs (si-CN).

PABPC1 is a poly (A)-binding protein family (PABPs) member. Over the past decade, many studies have demonstrated that viruses harness PABPs to favor viral RNA synthesis [33]. Hence, we investigated the effect of PABPC1 on RSV viral gene transcription and genome replication. We found that the RSV L and N mRNA levels were comparable between cells treated with si-CN and si-PABPC1 (Figure 3C,D). PABPC1 knockdown increased the accumulation of copies of the RSV genome in the cells (Figure 3E). Considering the result shown in Figure 3B, where PABPC1 knockdown led to a decrease in infectious particles, the data in Figure 3E suggested that PABPC1 knockdown likely impaired viral particle assembly.

In addition to the role of PABPC1 in mediating RNA synthesis [34,35], PABPC1 has also been reported to interact with virus proteins to control viral infections [23,36,37,38]. Therefore, we investigated whether any RSV proteins are associated with PABPC1. The IP results, shown in Figure 3F, demonstrated the interaction between PABPC1 and the RSV matrix protein (M).

### 3.3. The Effect of PABPC1 on RSV-Induced Cytokine/Chemokine Induction

**Figure 4:**
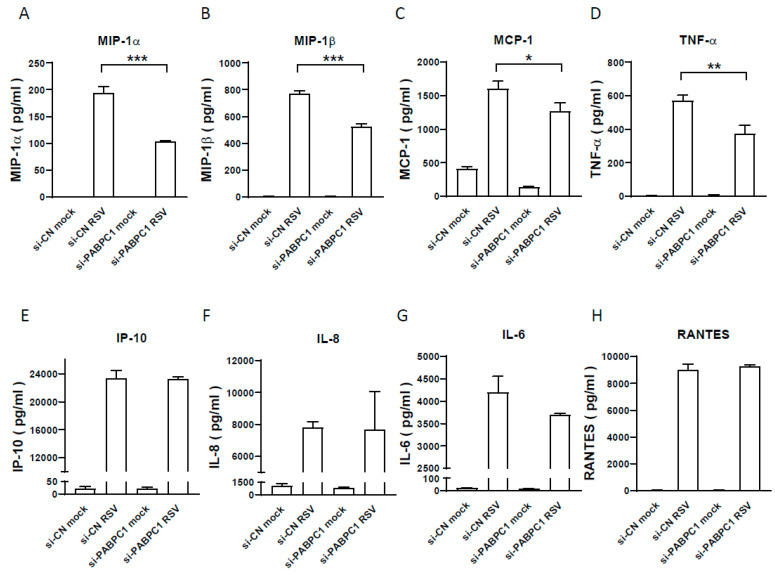
The impact of PABPC1 on RSV-induced cytokine/chemokine. The supernatants from the samples, shown in Figure 3, were subjected to a Bio-Plex multiplex immunoassay assay (Bio-plex). While MIP-1α (**A**), MIP-1β (**B**), MCP-1 (**C**), and TNF-α (**D**) were significantly reduced by si-PABPC1. Unchanged representative inflammatory mediators were also observed: IP-10 (**E**), IL-8 (**F**), IL-6 (**G**), and RANTES (**H**). * *p* ≤ 0.05, ** *p* ≤ 0.01, *** *p* ≤ 0.001, the fourth column compared to the second.

In response to RSV infection, the infected cells usually launch inflammatory or antiviral responses. To investigate whether PABPC1 knockdown also leads to changes in host responses to RSV in addition to viral replication, we used a Bio-plex multiplex system (Bio-Plex Pro Human Cytokine27-plex Assay, Bio-rad) to quantify the RSV-induced cytokines/chemokines in si-CN and si-PABPC1-treated cells. We found that a subset of RSV-induced inflammatory mediators, including MIP-1α, MIP-1β, MCP-1, and TNF-α, was reduced in PABPC1-deficient cells (Figure 4), suggesting that PABPC1 also partially controls inflammatory responses.

## 4. Discussion

tRFs have recently been found to have powerful regulatory functions, including gene expression, translation, and epigenetic control [39,40]. The aberrant expression of tRFs has been confirmed in multiple diseases, such as virus infection diseases [17,41,42,43], neurodegenerative diseases [15,16,44,45], and cancer [18,46,47,48]. In our previous series of studies, we found that tRF5-GluCTC was increased in RSV-infected patients and cells [17,21,42]. Viral-induced tRF5-GluCTC impairs APOER2 expression by matching to the 3′UTR of targets and facilitates viral replication [21]. In addition to the regulation of gene expression, tRFs can bind to RNA-binding proteins (RBPs) to exert biological functions [49]. In this study, we identified 31 RBPs that interacted with tRF5-GluCTC, including three RNA modification proteins (TRUB2, RPUSD4, and HARS2) (Table 2), which are aligned with tRNAs being enriched with chemical modification, and their modification status plays a vital role in determining their interaction with ribonuclease and the associated cleavage [50]. In our previous studies, we found that RSV induces change in the methylation and pseudouridine status of tRNA-GluCTC and that such changes are important in the cleavage of tRNA to generate tRF5-GluCTC [42]. tRFs also harbor multiple chemical modifications [51,52], and these modifications are important to tRFs’ function [53]. Therefore, our mass spectrometry discoveries regarding the association of TRUB2, RPUSD4, and HARS2 with tRF5-GluCTC implied their involvement in modifying tRF5-GluCTC/tRNA-GluCTC and their association with the function of tRF5-GluCTC.

tRFs have been reported to suppress global protein synthesis via their interaction with translation-related targets [53,54,55,55]. The studies conducted by Anderson lab found that ~30 nt-long tRF5-Ala and tRF5-Cys harbor a conserved motif of four to five guanine residues at their 5′ ends (TOG), and form intermolecular RNA G-quadruplexes, which competitively displace the translation–initiation factor eIF4F complex from m [7]G-capped mRNA [55,56]. In contrast to the long isoform TOG-containing tRF5s, the ~18-nt-long tRF5-Ala, tRF5-Cys, and tRF5-Val TOG-containing tRF5s, bearing pseudouridylation at the ψ8 position, interfere with translation–initiation complex assembly by binding with PABPC1 [53]. tRF5-GluCTC is 35nt in length and does not contain a TOG motif. However, we found and confirmed the binding of tRF5-GluCTC to PABPC1 (Figure 2), and our previous study identified the presence of pseudouridines at positions 19 and 20 in uninfected cells, with parts getting pseudouridylated by RSV infection [42]. In the future, we will determine the motifs and/or modifications contributing to tRF5-GluCTC-PABPC1 interaction in the context of RSV infection.

In addition to RNA modification proteins, we found 40S ribosomal protein S26 (RPS26) in the tRF5-GluCTC-protein complex (Table 2), suggesting that either the 5′-end of tRNA-GluCTC plays a role in ribosome binding during protein synthesis and/or that tRF5-GluCTC itself is involved in protein synthesis. Our previous studies revealed that cells treated with anti-tRF5-GluCTC or control oligos exhibit comparable protein synthesis in RSV infection [17], which makes the second possibility less likely. We also identified several mitochondrial proteins, including MRPL52, MRPL11, MRPL43, and MRM3, in the tRF5-GluCTC complex. It has been reported that 22 species of mitochondrial tRNAs, including tRNA-GluCTC, are encoded in mitochondrial DNA and are responsible for translating essential subunits of the respiratory chain complexes [57]. The importance of the 5′-end of mitochondrial tRNA-GluCTC in mitochondrial protein synthesis could also explain the binding of tRF5-GluCTC to these mitochondrial proteins. However, our previous studies showed that tRNA-GluCTC cleavage by RSV is controlled by a non-mitochondrial protein angiogenin (ANG) [17,58], which does not support the hypothesis that RSV-induced tRF5-GluCTC originates from the mitochondrial compartment. The interaction between tRF5-GluCTC and mitochondrial proteins likely represents an artificial binding due to the mixing of total cell lysates with synthesized tRF5-GluCTC. Therefore, we focus on the interaction between cytosolic proteins and tRF5-GluCTC.

**Figure 5:**
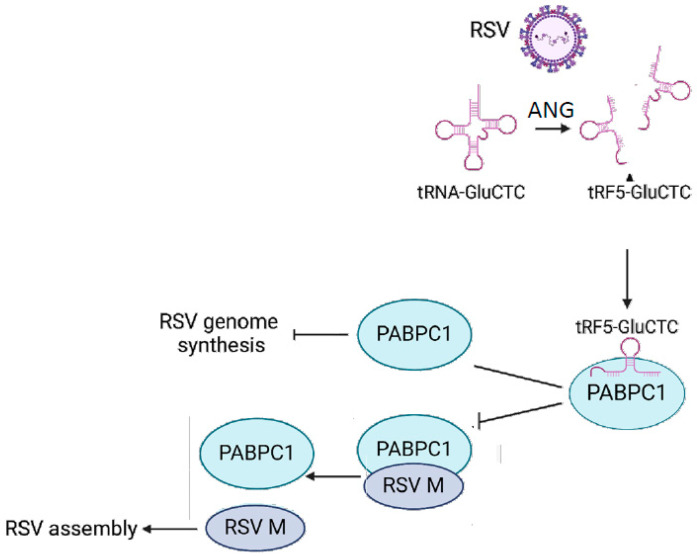
The model of PABPC1’s role in RSV infection. While the host PABPC1 is in a position to help the host control RSV genome synthesis, RSV induces tRF5-GluCTC to interfere with PABPC1 function. RSV infection induces cytosolic tRF5-GluCTC from its parent tRNA-GluCTC via angiogenin-mediated cleavage. The induced tRF5-GluCTC then binds to PABPC1, resulting in less PABPC1 being available to the RSV M protein, subsequently enhancing viral assembly.

As mentioned, PABPC1 plays a role in several viral infections [23,24,25,26]. Recently, PABPC1 has been reported to interact with the RSV protein M2-1, using an overexpression system [38]; this served as important motivation for us to explore the functions of PABPC1 in RSV infection. PABPC1 is important at multiple levels of mRNA regulation, including translational initiation, termination, stability, and mRNA-specific degradation [59,60]. In the context of viral infections, PABPC1 has been reported to be degraded or suppress other host proteins in viral infections [25,61,62]. Some viruses capture PABPC1 to help viral propagation [23,38,63]. Herein, our data did not support the changes in PABPC1 expression caused by RSV infection (Figure 2C). Based on the results of Figure 3, it seems that the host used PABPC1 to defend against viral genome synthesis (Figure 3E), but not against viral gene transcription (Figure 3C,D), as PABPC1 knockdown led to more viral genome copies without impacting the transcription of RSV nucleoprotein N or RNA-dependent RNA polymerase L protein. Despite the enhancedviral genome copies by PABPC1 downregulation, we found less viral progeny particles, indicating the interference of viral packaging or assembly (Figure 3B); this was an unexpected result, but can be explained by the interaction of PABPC1 with the RSV M protein (Figure 3E), an RSV protein known to regulate virion packaging and assembly [64]. Therefore, as speculated in Figure 5, while the host tried to use PABPC1 to defend against RSV genome synthesis, RSV, on the other hand, induces tRF5-GluCTC in the cytosolic compartment to interact with PABPC1 to reduce the interaction of PABPC1-M, subsequently making more M free and available for RSV assembly; this is a new evasion mechanism identified in this study. Overall, this study revealed that virus-induced tRF can bind host proteins to achieve replication.

## Figures and Tables

**Table 1:** Primer design for qRT-PCR and oligo information for pull-down experiments.

Target	Primer	Sequence (5′-3′)
PABPC1	Forward primer	GCCAGTACGCATCATGTGGTCTC
	Reverse primer	CATACAGTGCTTTATTATCAATGG
RSV N	RT primer	CTGCGATGAGTGGCAGGCTTTTTTTTTTTTAACTYAAAGCTC
	Forward primer	ACTACAGTGTATTAGACTTRACAGCAGAAG
	Reverse primer	CTGCGATGAGTGGCAGGC
RSV L	RT primer	CTGCGATGAGTGGCAGGCTTTTTTTTTTTTCATTATTCATTATG
	Forward primer	CTTACCTAAGTGAATTGTTAAACAGCTTGAC
	Reverse primer	CTGCGATGAGTGGCAGGC
RSV (-)	RT primer	CTGCGATGAGTGGCAGGCACTACAGTGTATTAGACTTRACAGCAGAAG
	Forward primer	GCATCTTCTCCATGRAATTCAGG
	Reverse primer	CTGCGATGAGTGGCAGGC
tRF5-GluCTC	RT primer	CGTCGGACTGTAGAACTCTCAAAGC
	Foward primer	TCCCTGGTGGTCTAGTG
	Reverse primer	CGTCGGACTGTAGAACTCTCAAAGC
Bio-tRF5-GluCTC		UCCCUGGUGGUCUAGUGGUUAGGAUUCGG-Biotin
Bio-tRF5-CN		AGGUCCAACUAAAUCACUAAUAAUAAACCGC-Biotin

**Table 2:** tRF5-GluCTC-bound proteins identified by proteomics. Selection criteria: the proteins were only detected in the Bio-tRF5-GluCTC complex (iBAQ > 0.005), or in the proteins in Bio-tRF5-GluCTC higher than Bio-tRF5-CN by ≥10-fold.

GeneID	GeneSymbol	iBAQ		Fold
		Bio-tRF5-CN	Bio-tRF5-GluCTC	Bio-tRF5-GluCTC/Bio-tRF5-CN
9406	ZRANB2		0.3415	
122704	MRPL52		0.0587	
55794	DDX28		0.0399	
65003	MRPL11		0.0383	
6231	RPS26	0.0011	0.0369	34.17
84545	MRPL43		0.0368	
84311	MRPL45		0.0350	
29088	MRPL15		0.0332	
2926	PAIP1		0.0324	
9130	FAM50A		0.0297	
1478	CSTF2		0.0243	
64928	MRPL14		0.0227	
3185	HNRNPF	0.0022	0.0220	10.00
51649	MRPS23		0.0210	
90480	GADD45GIP1		0.0198	
23438	HARS2		0.0193	
26986	PABPC1	0.0012	0.0183	15.69
28957	MRPS28	0.0017	0.0182	10.48
51335	NGRN		0.0178	
28998	MRPL13		0.0172	
55178	MRM3	0.0011	0.0169	15.54
29093	MRPL22		0.0164	
51263	MRPL30		0.0156	
28977	MRPL42		0.0106	
26995	TRUB2		0.0084	
27349	MCAT		0.0078	
51258	MRPL51		0.0070	
64969	MRPS5		0.0069	
51073	MRPL4		0.0069	
84881	RPUSD4		0.0057	
26024	PTCD1		0.0056	

## Data Availability

Available immediately after the publication.
